# Progress Toward Polio Eradication — Worldwide, 2014–2015

**Published:** 2015-05-22

**Authors:** José E. Hagan, Steven G.F. Wassilak, Allen S. Craig, Rudolf H. Tangermann, Ousmane M. Diop, Cara C. Burns, Arshad Quddus

**Affiliations:** 1 Epidemic Intelligence Service, CDC; 2 Global Immunization Division, Center for Global Health, CDC; 3 Polio Eradication Department, World Health Organization; 4 Division of Viral Diseases, National Center for Immunization and Respiratory Diseases, CDC

In 1988, the World Health Assembly of the World Health Organization (WHO) resolved to eradicate polio worldwide (1). Wild poliovirus (WPV) transmission has been interrupted in all but three countries (Afghanistan, Nigeria, and Pakistan). No WPV type 2 cases have been detected worldwide since 1999, and the last WPV type 3 case was detected in Nigeria in November 2012; since 2012, only WPV type 1 has been detected (2). Circulating vaccine-derived poliovirus (cVDPV), usually type 2, continues to cause cases of paralytic polio in communities with low population immunity (3). In 2012, the World Health Assembly declared global polio eradication “a programmatic emergency for global public health” (1), and in 2014, WHO declared the international spread of WPV to previously polio-free countries to be “a public health emergency of international concern” (4). This report summarizes global progress toward polio eradication during 2014–2015 and updates previous reports (5). In 2014, a total of 359 WPV cases were reported in nine countries worldwide (6). Although reported WPV cases increased in Pakistan and Afghanistan, cases in Nigeria decreased substantially in 2014, and encouraging progress toward global WPV transmission interruption has occurred. Overcoming ongoing challenges to interruption of WPV transmission globally will require sustained programmatic enhancements, including improving the quality of supplementary immunization activities (SIAs) to interrupt transmission in Afghanistan and Pakistan and to prevent WPV exportation to polio-free countries.

## Routine Poliovirus Vaccination Coverage

Estimated coverage in 2013 (the latest year for which complete data are available) among infants aged <1 year with 3 doses of oral poliovirus vaccine (OPV3) delivered through routine immunization programs was 90% in Afghanistan, 67% in Nigeria, and 66% in Pakistan, with substantial subnational variation. OPV3 coverage was 97% in the WHO Western Pacific Region, 96% in the European Region, 90% in the Region of the Americas, 82% in the Eastern Mediterranean Region, 77% in the African Region, and 76% in the South-East Asia Region (7).

## Supplementary Immunization Activities (SIAs)

In 2014, approximately 2.3 billion OPV doses were administered in 341 SIAs in 45 countries (Table 1), including 135 national immunization days, 147 subnational immunization days, 18 child health days, and 41 large-scale door-to-door immunization campaigns in areas where WPV was known or suspected to be circulating (“mopping-up” activities). One billion of the doses administered were trivalent (containing OPV types 1, 2, and 3), 1.1 billion were bivalent (types 1 and 3), and 79 million were monovalent type 1 OPV doses. SIAs in Afghanistan and Pakistan included those that were focused on children at border crossings from Pakistan, at borders of inaccessible districts, and at camps for refugees and internally displaced persons. In Nigeria, a national policy was adopted to respond to any new WPV case with multiple targeted SIAs.

## Poliovirus Surveillance

Polio cases caused by WPV and cVDPV are detected through surveillance for cases of acute flaccid paralysis (AFP) among children aged <15 years, followed by testing of stool samples at a WHO-accredited laboratory in the Global Polio Laboratory Network (8). Surveillance performance is measured using two principal indicators: the rate of nonpolio AFP detected,* and the percentage of adequate stool samples collected.† Among the 29 countries reporting either WPV or cVDPV cases during 2010–2014, a total of 21 (72%) met both surveillance performance indicators at the national level in 2014. Although the polio-endemic countries met both indicators, review of epidemiologic, environmental, and other virologic evidence revealed important surveillance gaps in all three countries (8).

## Reported Poliovirus Cases

**FIGURE 1:**
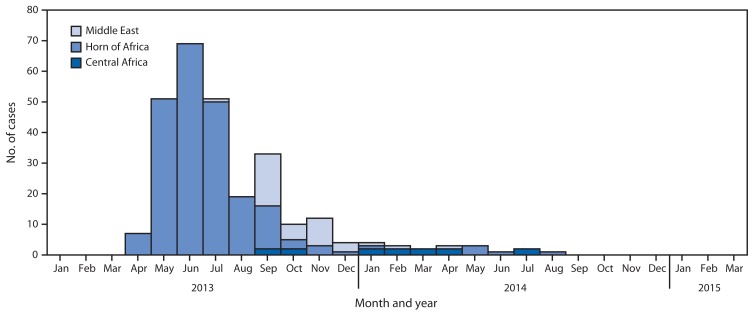
Number of cases of wild poliovirus type 1 in countries with recent polio outbreaks, by territory* — January 1, 2013–March 30, 2015 * Central Africa (Cameroon and Equatorial Guinea), Horn of Africa (Ethiopia and Somalia), and Middle East (Iraq and Syria).

During 2014, total of 359 WPV cases were identified (Figure 1); 306 (85%) were detected in Pakistan, 28 (8%) in Afghanistan, 6 (2%) in Nigeria, and 19 (5%) were in outbreaks following importation into previously polio-free countries in Central Africa (Equatorial Guinea and Cameroon), the Horn of Africa (Somalia and Ethiopia), and the Middle East (Iraq and Syria). During January 1–March 30, 2015, the low poliovirus transmission season, as of May 5, a total of 23 cases had been reported worldwide (22 from Pakistan, and one from Afghanistan). No cases were reported in nonendemic countries to date in 2015, compared with nine cases in five previously polio-free countries reported during the same period in 2014 (Table 2).

### Countries with endemic polio

**FIGURE 2:**
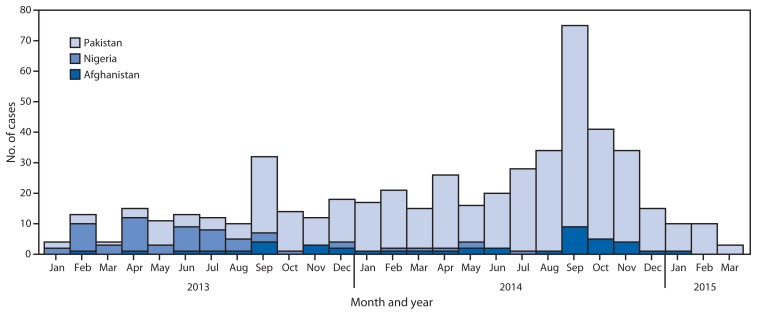
Number of cases of wild poliovirus type 1 among countries with endemic poliovirus transmission, by country — January 1, 2013–March 30, 2015

Nigeria reported six WPV cases in five districts in 2014, compared with 53 cases in 30 districts in 2013 (Figure 2). No WPV cases have been detected in Nigeria since July 2014, although cVDPV type 2 (cVDPV2) cases did increase, from four cases in 2013 to 30 cases in 2014, all in northern states. One WPV case was an orphan virus, indicating less than the expected genetic linkage to other circulating viruses, and suggesting possible gaps in AFP surveillance (9). Security concerns continue to restrict access by vaccination personnel to some northeastern areas and limit the ability to detect cases in these regions; however, 100% of local government areas met both AFP surveillance quality indicators in 2014.

In Afghanistan, the number of reported WPV cases doubled in 2014, to 28 cases in 19 districts, compared with 14 cases in 10 districts the preceding year. In 2014, 46% of cases were reported from Kandahar province; most other cases were reported from provinces neighboring the Pakistan Federally Administered Tribal Areas (FATA). All but four cases in 2014 (86%) were genetically linked to WPV importation from Pakistan. Three cases in 2014 were caused by orphan viruses, including one case of indigenous Afghanistan WPV, suggesting ongoing, undetected, low-level transmission and gaps in surveillance (10). No cVDPV has been detected in Afghanistan since early 2013. During January 1–March 30, 2015, one WPV case was detected, compared with four cases during the same period in 2014.

The largest increase in reported WPV cases in polio-endemic countries in 2014 occurred in Pakistan, where 306 cases were reported in 44 districts, a 230% increase in cases and a 91% increase in affected districts compared with 2013. During January 1–March 30, 2015, a total of 22 WPV cases were reported, compared with 59 cases reported during the same period in 2014. Reported cVDPV2 cases also decreased, from 48 cases in 2013 to 21 in 2014. Because of the ongoing threat of violence against polio workers, SIAs continued to be suspended or abbreviated in 2014 and 2015 in areas of Pakistan, including parts of Karachi, Peshawar, and FATA. During June 2012–June 2014, vaccination campaigns were banned by local governmental authorities in specific parts of FATA (North Waziristan), leaving an estimated 300,000 children aged <5 years inaccessible to vaccination teams. In 2014, 56% of all WPV cases reported from Pakistan were in persons who had received no doses of OPV, compared with no such WPV cases in Nigeria and 18% of all WPV cases in Afghanistan. A military operation in Waziristan in June 2014 was followed by improved access to that area during SIAs; the campaign was preceded by movement of large numbers of the resident population into surrounding safer areas of Pakistan and into Afghanistan, including approximately 250,000 children aged <5 years. Vaccination posts were arranged along transit routes, creating an opportunity for the vaccination of 550,000 children of all ages.

### Outbreaks in polio-free countries

In 2014, a total of 19 WPV cases were reported in six previously polio-free countries, a 93% decrease from 2013, when 256 WPV cases were reported in five polio-free countries. A large outbreak in the Horn of Africa following an importation of WPV of Nigerian origin accounted for 54% of WPV cases globally in 2013; the most recent case related to this outbreak occurred in Somalia on August 11, 2014. In late 2013 and early 2014, an outbreak affected Cameroon and Equatorial Guinea in Central Africa after a WPV importation of Nigerian origin. Onset of the most recent outbreak-related case was on July 9, 2014, in Cameroon. An exportation of WPV from Pakistan led to an outbreak including 36 cases in Syria and two in Iraq during 2013–2014; the most recent case related to this outbreak occurred on April 7, 2014, in Iraq.

What is already known on this topic?

Wild poliovirus (WPV) transmission now remains endemic only in Afghanistan, Nigeria, and Pakistan. During 2013–2014, outbreaks resulting from importation of WPV from those three countries occurred in eight previously polio-free countries in three world territories, threatening the progress made to date in achieving polio eradication.

What is added by this report?

In 2015, all three of the regional polio outbreaks in 2014 appear controlled, and reported WPV cases have decreased worldwide. No new cases have been detected in Nigeria since July 2014. However, transmission in Pakistan and Afghanistan continue in 2015, and control efforts are challenged by ongoing areas of insecurity.

What are the implications for public health practice?

Polio eradication appears increasingly feasible in the near future, bolstered by possible elimination of endemic WPV transmission from Nigeria and interruption of all the 2013–2014 outbreaks. The recent gains in polio control must build on continued coordinated commitment to improve childhood immunization in areas with low population immunity, strengthen acute flaccid paralysis surveillance, and use innovative strategies to access populations with supplemental immunization activities in the face of complex security and political challenges.

### Discussion

Four of six WHO regions have been certified as free of indigenous WPV, and endemic transmission of WPV continued in only three countries in 2014. In 2013, the global polio eradication effort suffered setbacks with outbreaks in the Horn of Africa, Central Africa, and the Middle East; however, significant progress was made in 2014 in response to all three outbreaks. Nonetheless, the affected regions remain vulnerable to WPV re-importation from endemic areas and to low-level, undetected WPV circulation. Continued response activities are needed in these regions to further strengthen AFP surveillance and eliminate immunity gaps through high-quality SIAs and strong routine immunization programs.

Progress in Nigeria since 2012 has brought the goal of interrupting the last known chains of indigenous WPV transmission in Africa within reach. Elimination of all poliovirus transmission in Nigeria in the near term is feasible, through intensified efforts to 1) interrupt cVDPV2 transmission, 2) strengthen routine immunization services, and 3) increase access to children in insecure areas. Similar efforts should be implemented in all countries in Africa, where 9 months have passed without a reported WPV case, and 6 months have passed since the last reported cVDPV2 case. Undetected circulation or re-importation of WPV into vulnerable countries such as those affected by the Ebola epidemic in West Africa, which suffered damage to routine health systems and deferment of polio SIAs, threatens recent progress in Africa.

Most (86%) WPV cases in Afghanistan in 2014 resulted from importation from Pakistan; however, the detection of orphan viruses highlights the need to strengthen the quality of both polio vaccination and AFP surveillance (10). Efforts are also needed to increase population immunity by intensifying routine polio immunization activities to ensure high coverage among infants with at least 3 OPV doses.

Recent challenges to the secure operation and public acceptance of the polio eradication program in Pakistan are unprecedented (10). Although poliovirus transmission has been concentrated primarily in the FATA region of northwest Pakistan, transmission has continued in the greater Karachi area, and WPV cases have been reported from all major Pakistan provinces. Successful efforts to enhance security to protect health workers and increase public demand for vaccination are urgently needed.

The recent gains in control and elimination of poliovirus transmission globally must be maintained and built upon through innovative strategies to access populations during SIAs in areas with complex security and political challenges, improve AFP surveillance, and strengthen routine immunization. With the progress achieved in 2014 to interrupt endemic WPV transmission in Nigeria and polio outbreaks in Africa and the Middle East, permanent interruption of global poliovirus transmission appears possible in the near future, provided that similar progress can be made in Afghanistan and Pakistan; progress there would also reduce the risk for future importation-related outbreaks in polio-free countries.

## Figures and Tables

**TABLE 1:** Number of SIAs conducted and number of OPV doses administered, by WHO region — 2013–2014

WHO region	2013		2014	
	
	SIAs	OPV doses	SIAs	OPV doses
AFR	154	853,508,010	142	775,972,255
AMR	2	24,502,802	0	0
EMR	114	561,943,748	183	639,908,596
EUR	2	3,118,271	8	6,351,137
SEAR	10	872,106,871	6	800,605,667
WPR	1	361,446	2	32,827,615
**Overall**	**283**	**2,315,541,148**	**341**	**2,255,665,270**

**Abbreviations:** OPV = oral poliovirus vaccine; SIAs = supplementary immunization activities; WHO = World Health Organization.

**Region abbreviations:** AFR = African Region; AMR = Region of the Americas; EMR = Eastern Mediterranean Region; EUR = European Region; SEAR = South-East Asia Region; WPR = Western Pacific Region.

**TABLE 2:** Number of reported poliovirus cases, by country — worldwide, January–March 2014 and 2015*

Country	2014 (January–December)		2014 (January–March)		2015 (January–March)	
		
	WPV	cVDPV	WPV	cVDPV	WPV	cVDPV
**Countries with endemic polio**						
Afghanistan	28	0	4	0	1	0
Nigeria	6	30	2	2	0	0
Pakistan	306	21	59	10	22	0
**Total**	**340**	**51**	**65**	**12**	**23**	**0**
**Countries without endemic polio**						
**Horn of Africa outbreak**						
Somalia	5	0	0	0	0	0
Ethiopia	1	0	1	0	0	0
**Central Africa outbreak**						
Equatorial Guinea	5	0	3	0	0	0
Cameroon	5	0	3	0	0	0
**Middle East outbreak**						
Iraq	2	0	1	0	0	0
Syria	1	0	1	0	0	0
**Other countries with reported cVDPV cases**						
South Sudan	0	2	0	0	0	0
Madagascar	0	1	0	0	0	0
**Total**	**19**	**3**	**9**	**0**	**0**	**0**
**Overall**	**359**	**54**	**74**	**12**	**23**	**0**

**Abbreviations:** cVDPV = circulating vaccine-derived poliovirus; WPV = wild poliovirus.

* Available data as of May 5, 2015.

## Footnotes

* Target = ≥1 case per 100,000 population aged <15 years for countries in WHO regions certified as polio-free; all other countries should achieve annual rates of ≥2.

† Target = ≥80%.

## References

[1] World Health Organization (2012). Poliomyelitis: intensification of the global eradication initiative.

[2] Kew OM, Cochi SL, Jafari HS (2014). Possible eradication of wild poliovirus type 3—worldwide, 2012. MMWR Morb Mortal Wkly Rep.

[3] Diop OM, Burns CC, Wassilak SG, Kew OM (2014). Update on vaccine-derived polioviruses— worldwide, July 2012-–December 2013. MMWR Morb Mortal Wkly Rep.

[4] World Health Organization (2015). Statement on the 4th IHR Emergency Committee meeting regarding the international spread of wild poliovirus.

[5] Moturi EK, Porter KA, Wassilak SGF (2014). Progress toward polio eradication—worldwide, 2013–2014. MMWR Morb Mortal Wkly Rep.

[6] World Health Organization (2015). Global Polio Eradication Initiative. Polio this week as of 5 May 2015.

[7] World Health Organization (2015). WHO vaccine-preventable diseases: monitoring system 2014 global summary.

[8] Porter KA, Diop OM, Burns CC, Tangermann RH, Wassilak SGF (2015). Tracking progress toward polio eradication—worldwide, 2013–2014. MMWR Morb Mortal Wkly Rep.

[9] Etsano A, Gunnala R, Shuaib F (2014). Progress toward poliomyelitis eradication—Nigeria, January 2013–September 2014. MMWR Morb Mortal Wkly Rep.

[10] Farag NH, Alexander J, Hadler S (2014). Progress toward poliomyelitis eradication—Afghanistan and Pakistan, January 2013–August 2014. MMWR Morb Mortal Wkly Rep.

